# Crimean-Congo hemorrhagic fever: serum neopterin levels and their relationship with clinical course

**DOI:** 10.1590/1806-9282.20250488

**Published:** 2026-01-09

**Authors:** Mustafa Usanmaz

**Affiliations:** 1 Samsun University, Samsun Training and Research Hospital – Samsun, Turkey.

**Keywords:** Crimean-Congo, Neopterin, Hemorrhagic fever, Immune response

## Abstract

**OBJECTIVE:**

Crimean-Congo hemorrhagic fever is a severe viral hemorrhagic disease transmitted by ticks and characterized by fever, malaise, and hemorrhages. This research was designed to measure serum neopterin concentrations in patients with Crimean-Congo hemorrhagic fever and to assess how these levels are associated with the disease's progression.

**METHODS:**

A total of 60 patients and 25 healthy controls, monitored at the Atatürk University Infectious Diseases Clinic from March 2009 to September 2010, were enrolled in the study. Serum neopterin concentrations were determined using an ELISA method, and statistical tests were conducted to explore their association with various clinical parameters.

**RESULTS:**

The results indicated a significant increase in serum neopterin levels in patients with Crimean-Congo hemorrhagic fever, and this rise was strongly linked to disease prognosis. Additionally, research has established that serum neopterin levels play a crucial role in determining patient outcomes, including survival and mortality.

**CONCLUSION:**

This study has demonstrated that neopterin levels can be used not only to track disease progression but also as a predictor of prognosis. Future research should delve deeper into the role of this biomarker in disease management.

## INTRODUCTION

Crimean-Congo hemorrhagic fever (CCHF) is a severe infectious disease transmitted by ticks and typically spreads from animals to humans through zoonotic transmission. First identified in Crimea in 1944, the disease was later detected in the Democratic Republic of the Congo, lending its name to both regions^1-3^. CCHF is caused by an RNA virus classified under the Nairovirus genus within the Bunyaviridae family. Humans contract this virus primarily through tick bites, and it has a wide geographical distribution. CCHF is prevalent in regions such as Asia, Africa, Eastern Europe, and the Middle East, and in Turkey, it occurs seasonally in the central and northeastern Anatolia regions^4^.

The infection can be transmitted between individuals via blood and other body fluids, putting healthcare workers at high risk^5^. With a mortality rate ranging from 3 to 30%, CCHF represents a major health concern, particularly in low-income regions^6^. While the precise mechanisms underlying the disease's pathogenesis remain unclear, the immune response plays a critical role in determining both disease severity and prognosis.

The clinical presentation of CCHF includes nonspecific symptoms such as sudden-onset fever, headache, fatigue, muscle pain, and bleeding tendencies. As the disease progresses, more severe complications such as petechial rashes, hepatosplenomegaly, and internal organ hemorrhages may arise^7^. Additionally, laboratory findings provide valuable insights into disease progression. Anomalies such as thrombocytopenia, leukocytosis, and elevated liver enzyme levels serve as key diagnostic indicators for CCHF^8^.

Neopterin is a biomarker secreted by macrophages and recognized as an indicator of cellular immune response^9^. Elevated neopterin levels play a pivotal role in assessing the severity and potential outcomes of viral infections, inflammatory diseases, and certain malignancies. Neopterin levels are particularly valuable in reflecting the immune response in viral hemorrhagic fevers^10,11^.

This study aims to investigate serum neopterin levels in CCHF patients and evaluate their relationship with the clinical course of the disease. Neopterin may serve as a potential biomarker for early diagnosis, disease monitoring, and assessing treatment response. Accordingly, the main goal is to reveal the association between neopterin levels and disease prognosis.

## METHODS

This research involved 60 patients diagnosed with CCHF at Atatürk University Infectious Diseases Clinic between March 2009 and September 2010, along with 25 healthy individuals serving as controls. Detailed medical histories were obtained from all participants, and demographic data were recorded. Clinical symptoms, physical examination findings, and laboratory results were documented. Patients were categorized into mild-moderate and severe groups based on the Swanepoel criteria^12^.

Inclusion criteria of patients and control group

- Age ≥18 years
- Laboratory-confirmed CCHF diagnosis
- Symptom onset within the last 7 days
- No prior antiviral or immunosuppressive treatment
- Informed consent obtained

Exclusion criteria of patients and control group

- Other active infections (e.g., HIV and hepatitis)
- Autoimmune or immunodeficiency disorders
- Current use of immunosuppressive or antiviral drugs
- Active malignancy
- Pregnancy or breastfeeding
- Severe liver or kidney failure
- Missing clinical/lab data
- No informed consent

Blood samples from patients were stored at −70°C to measure serum neopterin levels using the ELISA method (DRG Instruments, USA). The same procedures were applied to serum samples from the control group.

Data analysis was conducted with SPSS 20.0 software, and group differences were assessed using chi-square, Mann-Whitney U, and t-tests. The correlation between serum neopterin levels and clinical parameters was evaluated using Pearson correlation analysis.

## RESULTS

The findings of this study demonstrated that CCHF patients exhibited significantly higher serum neopterin levels compared to healthy individuals. Notably, 95% of patients experienced fever, 88% reported fatigue, and 75% had muscle pain (Table 1). Patients with hemorrhagic manifestations displayed a marked increase in serum neopterin levels (p<0.05).

**Table 1:** Demographic and clinical characteristics of patients.

Variable	CCHF patients (n=60)	Healthy controls (n=25)	p-value
Age (years)	45.3±12.5	42.1±10.8	0.234
Gender (male/female)	35/25	12/13	0.456
Fever (%)	95%	0%	<0.001
Fatigue (%)	88%	0%	<0.001
Muscle pain (%)	75%	0%	<0.001
Hemorrhagic symptoms (%)	60%	0%	<0.001

CCHF: Crimean-Congo hemorrhagic fever.

Physical examinations revealed a correlation between high neopterin levels and conditions such as hepatosplenomegaly and petechial rashes. A positive association was observed between white blood cell (WBC) counts and neopterin levels (p=0.006), while no conclusive statistical relationship was detected with platelet levels or liver function tests (Table 2).

**Table 2:** Correlation between neopterin levels and clinical parameters.

Parameter	Correlation coefficient (r)	p-value
White blood cell count	0.456	0.006
Platelet count	0.123	0.345
Liver enzyme levels	0.098	0.512

Comparing mild-moderate and severe cases, the mean serum neopterin level in mild-moderate patients was 16.368±0.450 ng/mL, whereas in severe patients, it was 17.500±0.260 ng/mL. Additionally, the comparison of surviving and deceased patients revealed a mean serum neopterin level of 21.200±14.000 ng/mL in survivors and 13.600±5.300 ng/mL in deceased patients, showing a notable statistical variance (p=0.021) (Table 3).

**Table 3:** Comparison of neopterin levels in Crimean-Congo hemorrhagic fever patients and healthy controls.

Group	Mean neopterin level (ng/mL)	Standard deviation	p-value
CCHF patients	18.500	2.300	<0.001
Healthy controls	5.200	1.100	

CCHF: Crimean-Congo hemorrhagic fever.

## DISCUSSION

This study demonstrated that serum neopterin levels were significantly elevated in CCHF patients compared to healthy controls, indicating a strong immune activation during the course of the disease. Neopterin, a known marker of cellular immune response, is primarily released by activated macrophages upon stimulation with interferon-gamma, reflecting Th1-type immune activity^13^.

Common symptoms such as fever, fatigue, and muscle pain observed in the majority of patients were consistent with findings from previous studies and remain important clinical clues in early CCHF diagnosis^14,15^.

Notably, bleeding symptoms—including epistaxis and gingival bleeding—were associated with significantly higher neopterin levels in our study, suggesting that immune activation may correlate with disease severity. This finding supports earlier reports that immune dysregulation plays a central role in the pathogenesis of viral hemorrhagic fevers^13,16^.

Hepatosplenomegaly and petechial rash were frequently detected on physical examination and were also accompanied by elevated neopterin levels. These manifestations, commonly linked to cytokine-mediated inflammation, highlight the possible contribution of macrophage activation and endothelial dysfunction in the clinical course of CCHF^17^.

Our laboratory data showed a significant association between elevated WBC count and increased neopterin levels, reinforcing neopterin's role as an immune activation marker^13^. However, we did not observe a meaningful correlation between neopterin and platelet counts or liver enzyme levels, indicating that neopterin may function as an independent prognostic biomarker, rather than one tied to organ-specific dysfunction.

Consistent with previous research findings, Ahlm et al. demonstrated that neopterin levels are essential in determining patient prognosis in European hemorrhagic fever cases^18^. Similarly, Baize et al. reported that lower neopterin levels in Ebola patients were associated with poorer prognoses^19^. Our study aligns with these findings, further supporting the role of neopterin as a key biomarker in CCHF.

Additionally, Yilmaz et al. noted similar trends in Turkish CCHF patients, further supporting the relevance of neopterin in disease progression^1^.

## CONCLUSION

This research demonstrated that serum neopterin levels may serve as a crucial biomarker for CCHF patients. The findings highlight that elevated neopterin levels signify a strong immune response, which plays a decisive role in disease progression and prognosis. Specifically, higher neopterin levels in survivors support the beneficial impact of an effective immune response on disease outcomes. Conversely, reduced neopterin levels in deceased patients may indicate an inadequate immune response.

Our research revealed a notable association between WBC count, disease severity, and serum neopterin levels, suggesting that neopterin levels could be used as a prognostic indicator. However, no meaningful statistical relationship was identified among other laboratory parameters and neopterin.

Incorporating neopterin levels into diagnostic, prognostic, and therapeutic strategies for viral diseases like CCHF can enhance patient management. Broadening the application of this biomarker in medical settings could benefit healthcare professionals and patients alike. Further studies should focus on a more comprehensive analysis of neopterin levels in larger patient populations and explore their interaction with other biomarkers.

Future research should validate these findings in larger cohorts and assess the utility of neopterin in combination with other inflammatory or viral load markers.

Moreover, evaluating neopterin as a potential therapeutic target may contribute to developing innovative strategies for managing high-risk conditions such as CCHF.

## Data Availability

The datasets generated and/or analyzed during the current study are available from the corresponding author upon reasonable request.
